# Recombinant human fibronectin segment (rhFN_1024_) hydrogel carried hPDLSCs to repair diabetic trauma by activated NF-κB signaling pathway

**DOI:** 10.1093/rb/rbaf027

**Published:** 2025-05-15

**Authors:** Jianhang Cong, Yating Cheng, Tongtong Liu, Xiang Cai, Jiahui Xu, Rui Guo, Rongrong He, Qi Xiang

**Affiliations:** State Key Laboratory of Bioactive Molecules and Druggability Assessment, Jinan University, Guangzhou 510632, China; Institute of Biomedicine and Guangdong Provincial Key Laboratory of Bioengineering Medicine, Jinan University, Guangzhou 510632, China; Biopharmaceutical R&D Center of Jinan University, Guangzhou 510632, China; State Key Laboratory of Bioactive Molecules and Druggability Assessment, Jinan University, Guangzhou 510632, China; Institute of Biomedicine and Guangdong Provincial Key Laboratory of Bioengineering Medicine, Jinan University, Guangzhou 510632, China; Biopharmaceutical R&D Center of Jinan University, Guangzhou 510632, China; Guangdong Engineering Research Center of Chinese Medicine & Disease Susceptibility/International Cooperative Laboratory of Traditional Chinese Medicine Modernization and Innovative Drug Development of Chinese Ministry of Education (MOE)/Guangdong Province Key Laboratory of Pharmacodynamic Constituents of TCM and New Drugs Research, Jinan University, Guangzhou 510632, China; State Key Laboratory of Bioactive Molecules and Druggability Assessment, Jinan University, Guangzhou 510632, China; Guangdong Engineering Research Center of Chinese Medicine & Disease Susceptibility/International Cooperative Laboratory of Traditional Chinese Medicine Modernization and Innovative Drug Development of Chinese Ministry of Education (MOE)/Guangdong Province Key Laboratory of Pharmacodynamic Constituents of TCM and New Drugs Research, Jinan University, Guangzhou 510632, China; State Key Laboratory of Bioactive Molecules and Druggability Assessment, Jinan University, Guangzhou 510632, China; Institute of Biomedicine and Guangdong Provincial Key Laboratory of Bioengineering Medicine, Jinan University, Guangzhou 510632, China; Biopharmaceutical R&D Center of Jinan University, Guangzhou 510632, China; State Key Laboratory of Bioactive Molecules and Druggability Assessment, Jinan University, Guangzhou 510632, China; Institute of Biomedicine and Guangdong Provincial Key Laboratory of Bioengineering Medicine, Jinan University, Guangzhou 510632, China; Biopharmaceutical R&D Center of Jinan University, Guangzhou 510632, China; Key Laboratory of Biomaterials of Guangdong Higher Education Institutes, Guangdong Provincial Engineering and Technological Research Center for Drug Carrier Development, Department of Biomedical Engineering, Jinan University, Guangzhou 510632, China; State Key Laboratory of Bioactive Molecules and Druggability Assessment, Jinan University, Guangzhou 510632, China; Guangdong Engineering Research Center of Chinese Medicine & Disease Susceptibility/International Cooperative Laboratory of Traditional Chinese Medicine Modernization and Innovative Drug Development of Chinese Ministry of Education (MOE)/Guangdong Province Key Laboratory of Pharmacodynamic Constituents of TCM and New Drugs Research, Jinan University, Guangzhou 510632, China; State Key Laboratory of Bioactive Molecules and Druggability Assessment, Jinan University, Guangzhou 510632, China; Institute of Biomedicine and Guangdong Provincial Key Laboratory of Bioengineering Medicine, Jinan University, Guangzhou 510632, China; Biopharmaceutical R&D Center of Jinan University, Guangzhou 510632, China

**Keywords:** recombinant human fibronectin peptide, integrin β1, advanced glycation end products, human periodontal ligament stem cells differentiation myofibroblasts, nuclear factor kappa-B signaling pathway

## Abstract

The accumulation of advanced glycation end products (AGEs) plays a crucial role in chronic inflammation and delayed wound healing in individuals with diabetes. In this context, fibronectin has been identified as a crucial protein that promotes the differentiation of human periodontal ligament stem cells (hPDLSCs) into myofibroblasts, which play a vital role in the repair of diabetic skin ulcers. This process is intimately associated with the integrin β1 receptor and the NF-κB signaling pathway, both crucial for cellular responses to fibronectin. To validate our hypothesis, we expressed rhFN_1024_, a recombinant protein containing the integrin β1 affinity-binding domain from human fibronectin segments 12–14. This protein was used to formulate a hydrogel for hPDLSCs. rhFN_1024_'s binding affinity to integrin β1 was confirmed by molecular docking and the cellular thermal shift assay (CETSA). We developed *sh-ITGB1*-hPDLSCs with stable ITGB1 knockdown using *shRNA-ITGB1* and compared their proliferation, migration and adhesion to wild-type hPDLSCs. Morphological changes were observed via SEM, and α-SMA expression levels were measured in AGEs-damaged hPDLSCs. We created full-thickness wound models in diabetic mice to assess pharmacodynamics. The study showed that rhFN_1024_ stimulated hPDLSCs differentiation into myofibroblasts by boosting ITGB1 expression. rhFN_1024_ also reduced AGEs' negative effects on hPDLSCs, as seen through SEM analysis and α-SMA levels. In full-thickness wound models, hPDLSCs and rhFN_1024_ accelerated re-epithelialization and collagen synthesis. rhFN_1024_ is proposed to interact with the ITGB1 receptor on hPDLSCs, activating the NF-κB pathway to neutralize AGEs-induced pro-inflammatory cytokines. This study suggests rhFN_1024_ as a potential biomedical material for tissue repair.

## Graphical abstract

**Figure:**
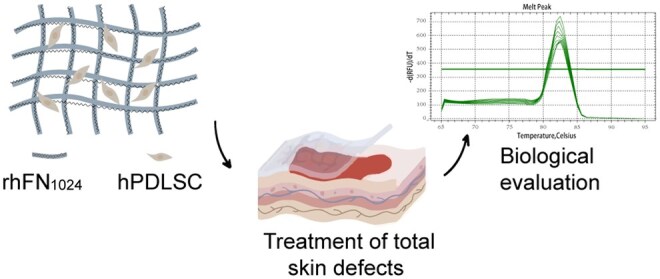

## Introduction

Diabetes is a prevalent metabolic disorder in the 21st century. Diabetic ulcer is one of its most influential complications. Its global costs exceed 760 billion dollars, accounting for 10% of annual healthcare expenditure for adults. By 2045, it is expected to affect over 700 million people [1, 2]. Advanced glycation end products (AGEs), byproducts of glycation reactions, can still form even with controlled blood sugar. Their accumulation is linked to chronic diseases such as diabetic complications, cardiovascular diseases and aging. Diabetic trauma involves hyperglycemia and peripheral vascular occlusion caused by AGEs [3–5]. The occurrence and progression of diabetes and inflammation are intricately linked to the accumulation of AGEs, which activate various signaling pathways, such as nuclear factor kappa-B (NF-κB), by interacting with the receptor for advanced glycation end products (RAGEs). This activation exacerbates the inflammatory response and results in the release of pro-inflammatory cytokines such as tumor necrosis factor-alpha (TNF-α) and interleukin-6 (IL-6). AGEs can cause endothelial dysfunction and vascular damage, increase the occurrence and progression of vascular inflammation, and further aggravate inflammatory responses in diabetes. These effects delay or prevent wound healing in diabetics. Healing diabetic wounds is particularly challenging due to chronic hyperglycemia and AGEs formation.

Clinical management of diabetic trauma faces challenges, encouraging novel approaches to enhance tissue regeneration. Mesenchymal stem cells (MSCs) play a crucial role in modulating wound healing and tissue regeneration processes [6–8]. In diabetic rat wound models, MSCs administration increased angiogenesis, accelerating wound healing and promoting tissue regeneration [9]. Human periodontal ligament stem cells (hPDLSCs), undifferentiated MSCs in periodontal membrane tissue, are used in regenerative medicine due to their availability and ethical benefits [10]. Recent studies have demonstrated that surface modification of biomaterials—such as using sandblasted/etched titanium disks—significantly enhances VEGF/VEGF-R and RUNX2 expression in periodontal ligament stem cells, thereby promoting their osteogenic and angiogenic differentiation. Moreover, the nuclear translocation of PKCα plays a crucial role in driving the neurogenic differentiation of these cells, offering promising new avenues for cell-based therapies [11, 12]. hPDLSCs exhibit higher growth and proliferation potential than human bone marrow mesenchymal stem cells (BMSCs), making them a promising new research focus [13]. We used hPDLSCs to repair alveolar bone and oral soft tissue with effective results [14, 15]. Numerous studies highlight hPDLSCs' therapeutic potential in regenerative medicine.

However, there are still shortcomings and restrictions to the clinical application of hPDLSCs. Using a scaffold as an effective carrier for hPDLSCs would improve their survival and maintain their stemness, thus increasing clinical efficacy. Fibronectin (FN), an extracellular matrix (ECM) protein, plays a crucial role in regulating cell adhesion, spreading, proliferation and apoptosis, and is utilized in wound healing [16]. FN interacts with cell surface integrins, including α5β1, α5β3 and α5β6 [17]. FN, unlike collagen, is a well-characterized large glycoprotein that promotes cell adhesion within the ECM. rhFN_1024_ can bind to integrin β1 (ITGB1) receptors on the cell surface, providing support and signals for hPDLSC adhesion and proliferation, thereby promoting cell survival and functionality [18]. Single-cell RNA sequencing has revealed that hPDLSCs express characteristic myofibroblast-expressed genes, indicating that hPDLSCs could differentiate into myofibroblasts mediated by integrin β1 [19]. Consequently, there is significant interest in creating novel proteins with specific ECM ligands, inspired by the functional traits of key ECM structural proteins.

## Materials and methods

### Culture of cells

hPDLSCs were isolated from the middle third of tooth root tissues of healthy individuals aged 15–20 years at the First Affiliated Hospital of Jinan University. The Ethics Committee of Jinan University (Guangdong, China) approved all experimental protocols (approval number JNUKY-2023-013).

Human Embryonic Kidney 293 (HEK293) cells were kept in a high-glucose DMEM medium supplemented with 10% fetal bovine serum (FBS). Both cell lines were incubated at 37°C with 5% CO_2_ and 95% humidity.

### Transfecting plasmid DNA into HEK 293T and hPDLSCs

HEK 293T cells were transfected with plasmids encoding pCMV-n-Flag-EGFP-rhFN_1024_ using Lipofectamine 3000.

hPDLSCs were transfected with psi-LVRU6GP-*sh-ITGB1* using lentiviral packaging reagents (FuNeng Gene Biotechnology Co., Ltd, China) (Supplementary Table S1).

### Construction and identification of rhFN_1024_

The gene encoding rhFN_1024_ (ID: M10905.1, 384–1194 nt) was transferred into the pET-20b (Invitrogen, Guangzhou, China) expression vector, resulting in the recombinant plasmid pET-20b-rhFN_1024_. Subsequently, *Escherichia coli BL21 (DE3)* and *pLysS* (Invitrogen, Guangzhou, China, ATCC^®^ BAA-1025^™^) were transformed with this plasmid. After establishing the optimal expression conditions, large-scale production of rhFN_1024_ was conducted in a 15-l fermenter. The rhFN_1024_ protein was subsequently purified through a multi-step process involving Ni Sepharose 6 Fast Flow affinity chromatography followed by cation exchange chromatography. The identification of rhFN_1024_ utilized polymerase chain reaction, Western blot, gel electrophoresis and circular dichroism (CD) spectrum (Photophysics Ltd, Leatherhead, Surrey, UK) analysis were employed.

### Molecular docking prediction of rhFN_1024_ with ITGB1

ITGB1 (PDB ID: 4WK0) and rhFN_1024_ (PDB ID: 3R8Q) were retrieved and downloaded from the PDB Protein Structure Database. To further explore the relevant molecular interactions, the PatchDock server (http://bioinfo3d.cs.tau.ac.il/PatchDock) was used to simulate the interactions between rhFN_1024_ and ITGB1. The FireDock server (http://bioinfo3d.cs.tau.ac.il/FireDock) was used to present the docking results. Finally, the protein surface interactions were analysed using PyMOL v2.5.1.

### Cellular thermal shift assay

HEK 293T cells overexpressing EGFP-rhFN_1024_, ITGB1 and EGFP-rhFN_1024_@ITGB1 were collected using cell lysate. A 12 000 *g* centrifugation for 20 min was performed, and the supernatant was taken. The supernatants of each group were divided into 10 portions after BCA quantification, heat-treated at 37, 41, 45, 49, 53, 57, 61, 65, 69 and 73°C for 3 min, cooled to room temperature and separated into supernatants. The levels of integrin β1 (ABclonal Technology, China, Cat# A22599-PM) were detected by Western blotting.

### Assays for cell division, migration and adhesion

The effects of rhFN_1024_ on cells were demonstrated using proliferation, scratch assay, crystal violet staining and cytoskeleton staining, as described previously [20].

### SEM analysis

hPDLSCs and short hairpin RNA targeting integrin β1 in hPDLSCs (*sh-ITGB1*-hPDLSCs) were treated with AGEs at 50 μg/ml, either alone or alongside rhFN_1024_ at concentrations of 6 and 12 μg/ml, for durations of 4 or 8 days. The control group of hPDLSCs was not treated with AGEs or rhFN_1024_. The morphology of cells was observed through scanning electron microscopy (SEM) (SEM, XL30; Philips, Amsterdam, the Netherlands).

### Preparation of rhFN_1024_ hydrogel and physicochemical characterization

The hydrogel was prepared as described previously [21]. Briefly, 26% w/v Kolliphor^®^ P407 (P407, BASF, Germany, Cat# GNG23121B) was swollen with phosphate buffer solution (PBS) at 4°C to achieve a homogeneous Poloxamer 407 hydrogel (P407) solution, then stored at 4°C until use. To construct a P407/rhFN_1024_ hydrogel, rhFN_1024_ was dissolved in a small quantity of PBS. The P407 solution was stirred into the rhFN_1024_ solution at 4°C. To load hPDLSCs, hPDLSCs were resuspended in rhFN_1024_ PBS before use, then mixed with P407 solutions at 4°C.

The structures of the hydrogel were characterized by SEM (HITACHI S-4800), Fourier Transform Infrared (FTIR, PerkinElmer Spectrum 100 ATR-IR spectrometer, PerkinElmer, Waltham, MA) in conjunction with Spectrum Software version 6.3.1.0134 (PerkinElmer), CD Spectroscopy (Applied Photophysics, Leatherhead, UK) and Rheology.

### Biocompatibility test

The viability of cells was assessed using the Cell Counting Kit-8 (CCK-8) reagent. A 500 µl P407 hydrogel or P407/rhFN_1024_ hydrogel sample was added to a 24-well plate. Next, the L929 cell suspension was seeded into the wells of the 24-well plate, with 20 000 cells in each well. The cells were cultured for 72 h, and cell viability was evaluated by adding CCK-8 solution to each well. The absorbance was quantified using a microplate reader (SH1000, Corona, Japan) at a wavelength of 450 nm.

### *In vivo* studies on a diabetic mouse model with full-thickness skin defects

Male C57BL/6 mice aged 6–8 weeks and weighing 25–30 g were obtained from the Guangdong Medical Laboratory Animal Centre in China (certificate no. 44007200069979). The mice were maintained in a pathogen-free, controlled environment with a 12-h light/dark cycle and had *ad libitum* access to food and water for one week. This standard housing condition was maintained to ensure the well-being and health of the mice throughout the experimental period. The research was authorized by the Ethics Review Committee for Animal Experimentation at Jinan University (ethical review no. 20230510-0003). Mice were administered low-dose streptozotocin intraperitoneally for seven days to induce insulin deficiency and hyperglycemia. Mice with blood glucose levels exceeding 16.7 mmol/l were classified as diabetic. Mice were given low-dose streptozotocin injections for seven days to induce insulin deficiency and hyperglycemia. Mice were deemed diabetic if their blood glucose levels were above 16.7 mmol/l.

A 6 mm punch biopsy tool was used to create wounds on the mice's backs, which were then randomly assigned to one of seven groups: control, vehicle, hPDLSCs (5 × 10^5^), rhFN_1024_ (200 μg/ml), rhFN_1024_ (400 μg/ml), hPDLSCs (5 × 10^5^)-rhFN_1024_ (200 μg/ml) (hPDLSCs@L-rhFN_1024_ group) and hPDLSCs (5 × 10^5^)-rhFN_1024_ (400 μg/ml) (hPDLSCs@H-rhFN_1024_ group). Each group was applied to the wounds, and wound healing was observed. Tissue samples were collected from mice sacrificed on days 3, 5, 7 and 14. The healing rate was calculated as:

$$\text{Healing}\;\text{rate}=\frac{\left(S_{0}-S_{t}\right)}{S_{0}}\times 100\%$$

where *S*_0_ represents the initial wound area and *S_t_* denotes the wound area on day *T*.

### Quantitative real-time (RT)-PCR

Total RNA was extracted with TRIzol and reverse transcribed using a high-capacity cDNA synthesis kit (Accurate Biotechnology, Guangzhou, China). RNA levels were quantified with SYBR^®^ Premix Ex Taq II (TaKaRa) on a CFX Connect reverse transcription machine (Bio-Rad) and analysed via the relative quantitation method.

### Western blot analysis

Western blotting (WB) was performed as previously described [22]. Alpha-Smooth Muscle Actin (α-SMA) antibody was obtained from BOSTER (USA) and used at a 1:1000 dilution, while vimentin antibody and ITGB1 were purchased from Bioss (China) and used at a 1:1000 dilution. Secondary antibodies were purchased from FUDE Biological Technology (China) and used at a 1:5000 dilution. Pierce ECL chemiluminescent substrates (Thermo Scientific, Waltham, MA) were used for blot development and detection.

### Immunohistochemical analysis

On the 14th day of the experiment, wound tissue samples from the mouse model were processed for immunohistochemical (IHC) analysis. The sections were treated to retrieve antigens, block endogenous peroxidase, permeabilize and block nonspecific binding sites. Antibodies against specific proteins including ITGB1 (1:200; Affinity), α-SMA (1:200; Affinity), NF-κB (1:200; Affinity), Protein Kinase B (AKT) (1:200; Affinity), Phosphoinositide 3-Kinase (PI3K) (1:200; Affinity) and Interleukin-1β (IL-1β) (1:200; Affinity) were applied and incubated overnight at 4°C. After washing with PBS, a secondary antibody was applied for 40 min. The sections were then stained with DAB and hematoxylin for visualization.

### Statistical analysis

The data were presented as the mean ± standard deviation (SD) of a minimum of three independent experiments and were statistically analysed using one-way analysis of variance with a Tukey honestly significant difference (HSD) test. A significance level of *P* < 0.05 was applied. Statistical analysis was performed using GraphPad Prism 8.0 software from GraphPad Software Inc., located in La Jolla, CA, USA.

## Results

### Construction, expression and identification of rhFN_1024_

**Figure 1.**
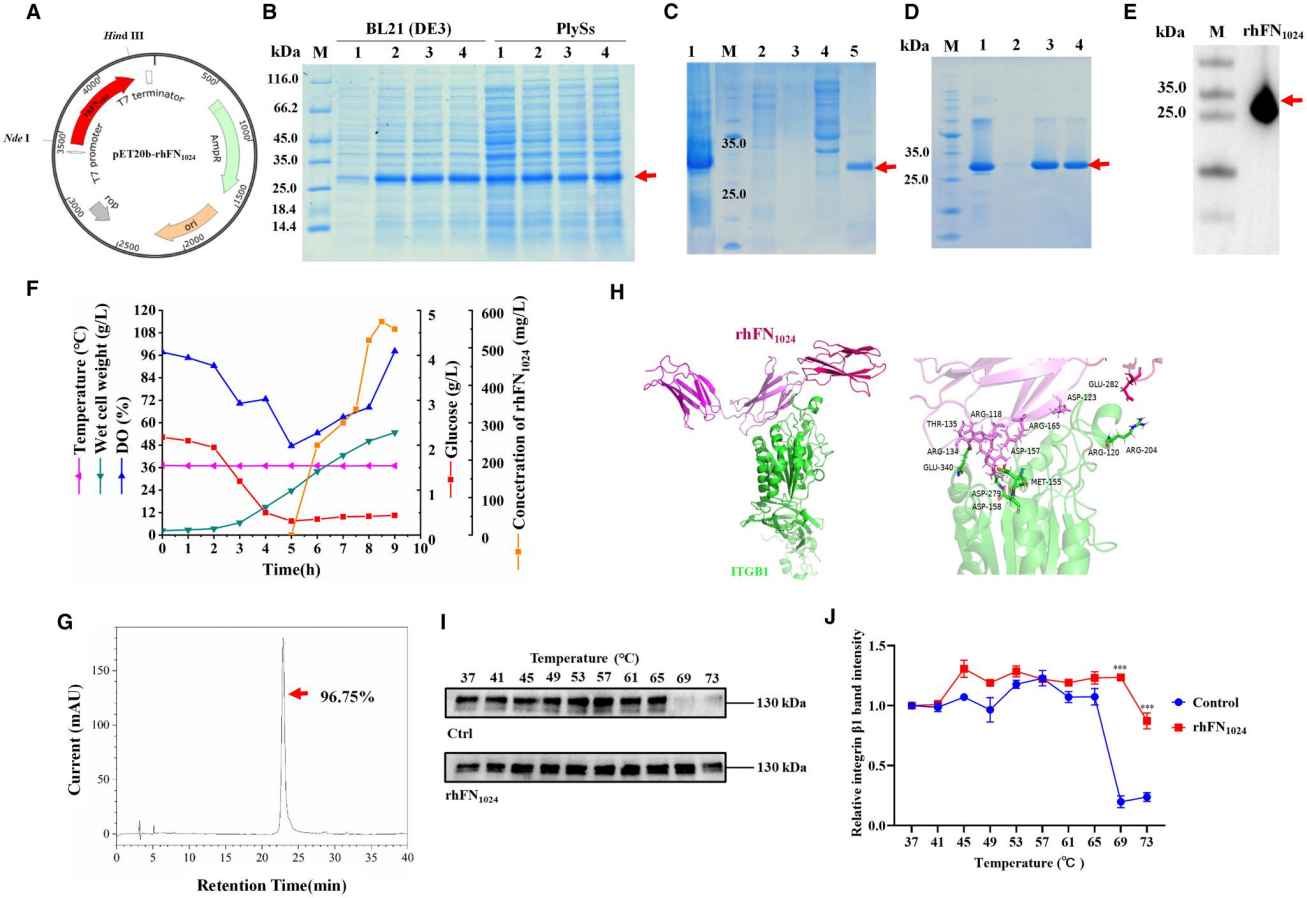
Construction, expression and identification of rhFN_1024_. (**A**) Recombinant pET20b-rhFN_1024_ plasmid profile. (**B**) SDS-PAGE analysis was conducted to assess rhFN_1024_ expression across various strains. M: molecular weight marker; lane 1: rhFN_1024_ expression before induction; lanes 2–4: rhFN_1024_ expression after induction. (**C**) Nickel column affinity chromatography. M: molecular weight marker; lane 1: rhFN_1024_ expression after induction; lane 2: flowthrough; lanes 3–5: supernatants eluted with 50, 100 and 200 mM imidazole, respectively. (**D**) Cationic affinity chromatography. M: molecular weight marker; lane 1: rhFN_1024_ after Ni column purification; lane 2: flowthrough; lanes 3–5: supernatants eluted with 150 mM NaCl. (**E**) Western blotting of rhFN_1024_. M: molecular weight protein markers; lane 1: rhFN_1024_ expression. (**F**) 15 l fermentation parameters of rhFN_1024_. (**G**) HPLC analysis of the purity of rhFN_1024_. (**H**) Molecular docking of rhFN_1024_ (PDB ID: 3R8Q) and ITGB1 (PDB ID: 4WK0). (**I**) The expression of ITGB1 with increasing temperature assessed by Western blotting analysis. (**J**) Semi-quantitative analysis of integrin β1 expression, measured using the ImageJ software. *n* = 3, mean ± SD, ***P < 0.001 vs. the control.

The coding sequence of rhFN_1024_ was integrated into the pET-20b vector to produce a recombinant plasmid designated as pET20b-rhFN_1024_ (Figure 1A). Then, pET20b-rhFN_1024_ was transformed into *E. coli BL21(DE3)* and *pLysS*. The highly expressed *BL21* and *pLysS* strains were screened out. In total, rhFN_1024_ accounted for approximately 40% of the total supernatant protein content in *BL21*, which was significantly higher than in *pLysS*, where it represented approximately 28% (*P* < 0.001) (Figure 1B). *E. coli BL21* was then selected for the large-scale production of rhFN_1024_ using a 15 l fermenter (Figure 1F). In addition, rhFN_1024_ was purified via Ni-NTA Sepharose affinity chromatography and cationic affinity chromatography (Figure 1C and D). Western blot analysis revealed a positive band at 30 kDa (Figure 1E), and further analysis using HPLC determined that the purity of rhFN_1024_ was above 96.75% (Figure 1G). The yield of rhFN_1024_ was approximately 2 g/l, which was higher than previously reported [20]. LC–MS/MS showed that the molecular weight of rhFN_1024_ was 30.36 kDa, which was consistent with its theoretical molecular weight (Supplementary Figure S1A and B).

### The interaction between rhFN_1024_ and ITGB1 and its impact on thermal stability

The 3D image (Figure 1H) shows that rhFN_1024_ and ITGB1 formed a complex with an interaction area of 1020.6 Å^2^ and free energy of 0.7 ΔiG. More specifically, the 2D view shows a total of seven amino acid residues of rhFN_1024_ (Thr135, Arg165, Arg134, Arg118, Glu282, Asp123 and Arg120) and seven amino acid residues of integrin β1 (Glu340, Asp279, Asp158, Met155, Arg204, Lys202 and Asp157) as the key sites involved in protein–protein binding. These amino acid residues interacted mainly through hydrogen and salt bonds (Supplementary Figure S1C and D). To further evaluate whether rhFN_1024_ increases the stability of integrin β1 within cells, we overexpressed rhFN_1024_ in 293T cells (Supplementary Figure S2). Cellular thermal shift assay (CETSA) results indicated that rhFN_1024_, when bound to endogenous ITGB1, enhanced its thermal stability (Figure 1I and J). Overall, rhFN_1024_ binding to endogenous ITGB1 improved its thermal stability.

### Physicochemical and biological characterization of hydrogel

**Figure 2.**
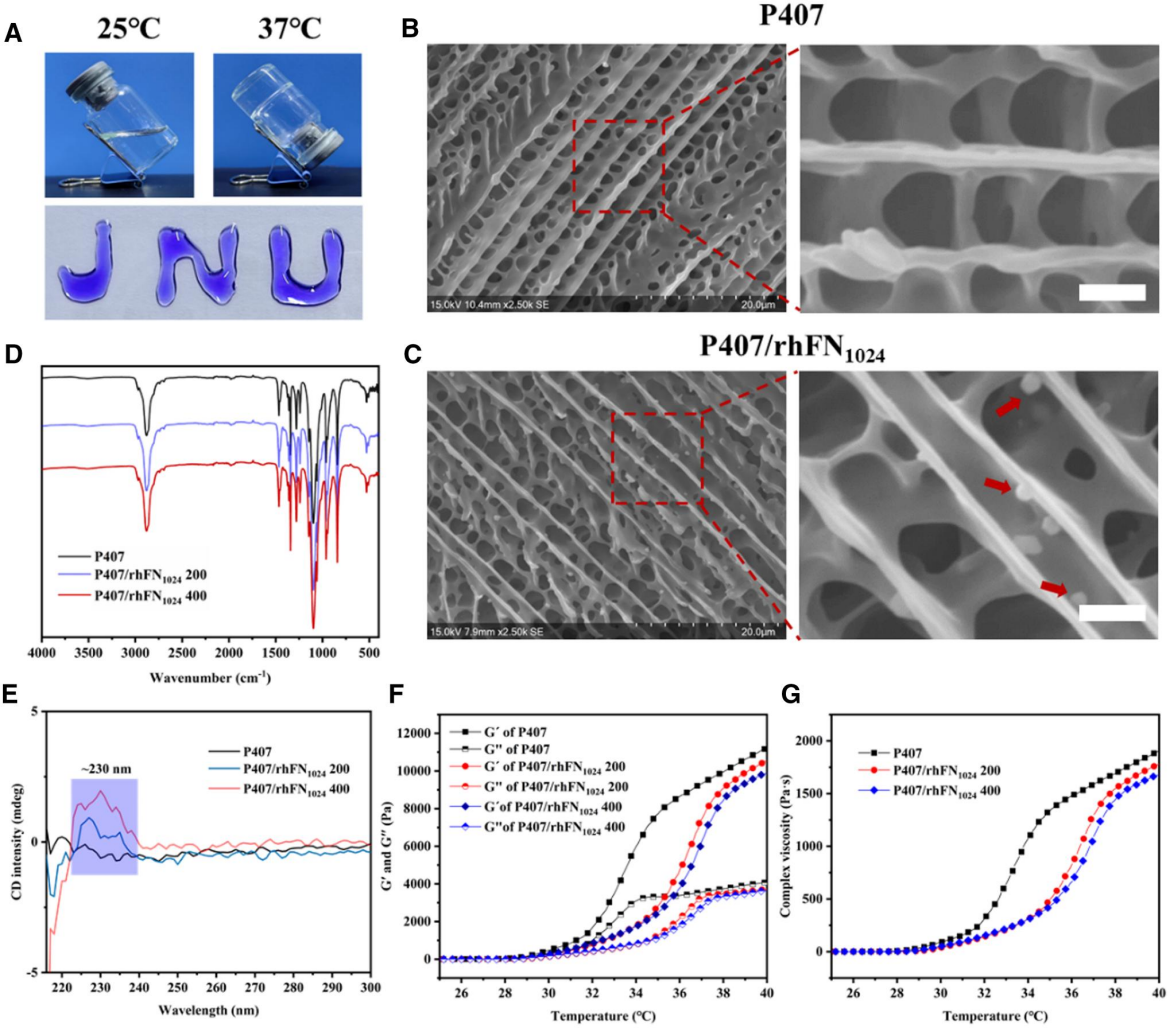
Analysis of P407/rhFN_1024_/hPDLSCs hydrogel. (**A**) Optical images of the hydrogel at different temperatures. (**B**) SEM images demonstrating the porous structure of P407 hydrogel (scale bar = 2 μm). (**C**) SEM images demonstrating the porous structure of P407/rhFN_1024_ hydrogel (scale bar = 2 μm). (**D**) FT-IR spectroscopy of the hydrogel. (**E**) CD spectra of hydrogel. (**F** and **G**) Rheological properties of hydrogel from 25 to 40°C.

As illustrated in Figure 2A, the P407/rhFN_1024_ hydrogel exhibits a liquid and nearly transparent state at low temperatures. Upon increasing the temperature from 25 to 37°C, the hydrogel transitions into a semisolid gel. Both P407 hydrogel and P407/rhFN_1024_ hydrogel exhibited an interconnected porous microstructure that was notably similar (Figure 2B and C). The presence of particles on the surface of the P407/rhFN_1024_ hydrogel was observed, which could be indicative of rhFN_1024_ particles. The loose and porous network structure exhibits good absorptivity and breathability, which facilitates the exchange of substances and promotes skin wound healing. As shown in Figure 2D, the results demonstrated that the FTIR spectra of P407 hydrogel and P407/rhFN_1024_ hydrogel exhibited near-identical characteristics, indicating that the introduction of rhFN_1024_ did not result in the formation of a novel chemical bond. rhFN_1024_ was incorporated into the P407 hydrogel in a physically mixed manner. The P407/rhFN_1024_ hydrogel was investigated by CD spectroscopy. The results demonstrated that the P407/rhFN_1024_ hydrogel exhibited a positive band at 230 nm (Figure 2E), indicating the presence of an irregularly curled structure. This evidence substantiates the incorporation of rhFN_1024_ into the P407 hydrogel. The rheological properties of the P407/rhFN_1024_ hydrogel are shown in Figure 2F. With the increase in temperature, at 25–29°C (due to the loss of moisture caused by hot air heating, which lowers the gelation temperature), the energy storage modulus *G*′ is equal to the energy dissipation modulus *G*′, indicating that the temperature-sensitive gel undergoes the phenomenon of gelling. Thereafter, the energy storage modulus *G*′ becomes greater than the energy dissipation modulus *G*′, indicating that the gel solution completes the transition from the liquid state to the semisolid state.

As illustrated in Figure 2G, the change curve of complex viscosity over time reveals that when the temperature is below 29°C, the hydrogel system exists in a liquid state, exhibiting a relatively smooth increase in complex viscosity. However, when the temperature exceeds 31°C, the complex viscosity of the hydrogel system increases at a faster rate, indicating a gradual transition into a semisolid gel state.

Medical dressings are products that directly contact the tissue cells of a wound and must be nonirritating, nontoxic and nonsensitizing to the human body. Assessing material biocompatibility is crucial in biomedical material research and development. L929 cells were used in this assay because they play an integral role in the wound healing process. The CCK-8 assay demonstrated that both the P407 hydrogel and the P407/rhFN_1024_ hydrogel did not significantly affect L929 cell proliferation (Supplementary Figure S3).

### Integrin β1 shRNA transfection suppressed the effects of rhFN_1024_ on hPDLSCs

**Figure 3.**
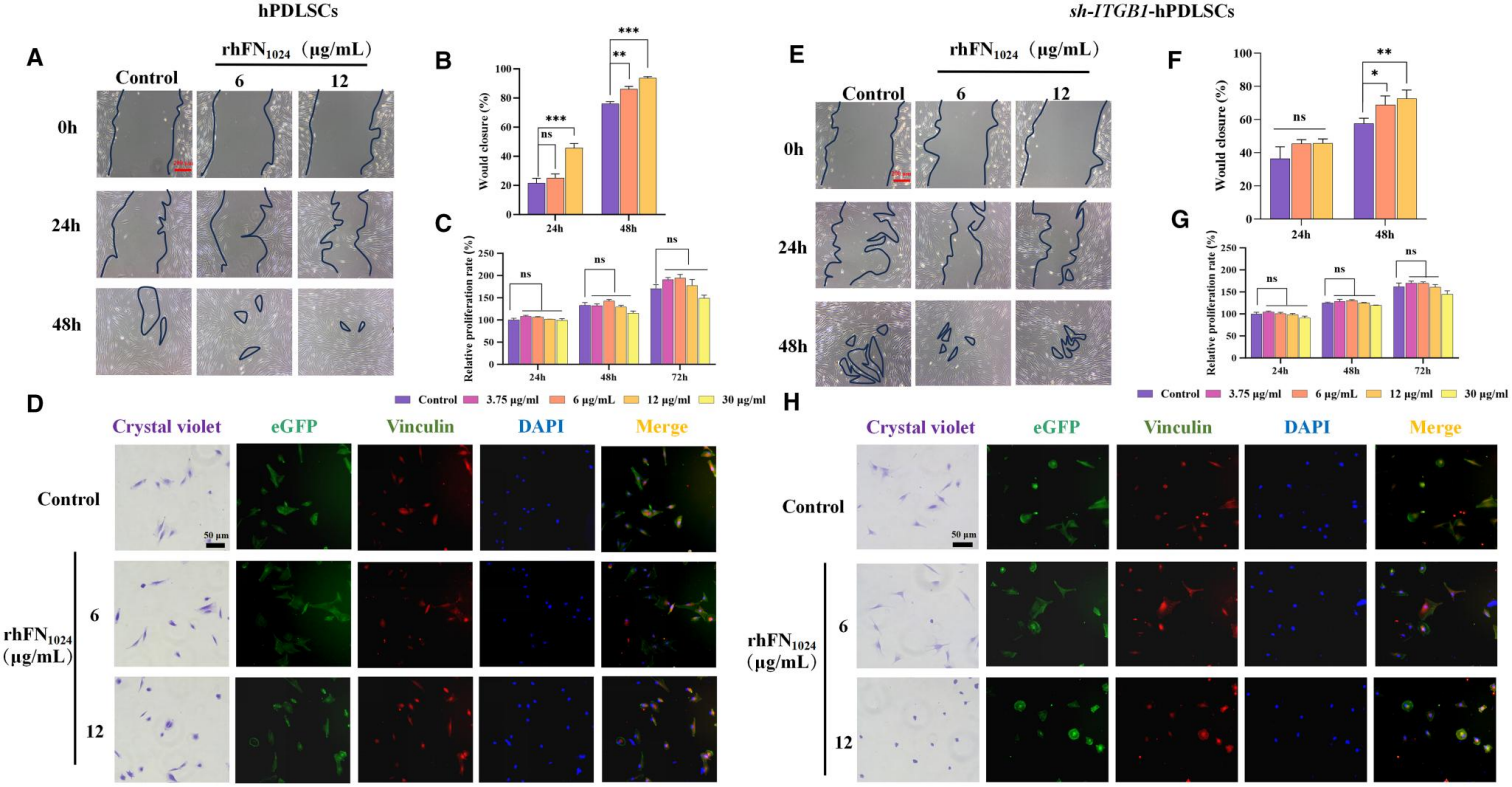
Impact of rhFN_1024_ on hPDLSCs and *sh-ITGB1*-hPDLSCs proliferation, migration and adhesion. (**A**) Migration of hPDLSCs cells cultured on rhFN_1024_ was observed at 0, 24 and 48 h (scale bar = 200 μm). (**B**) Semi-quantitative analysis of the gap area of hPDLSCs cells cultured on rhFN_1024_. (**C**) CCK-8 was used to detect the effects of different concentrations of rhFN_1024_ on the proliferation of hPDLSCs after 24, 48 and 72 h. (**D**) Determination of the adhesion of rhFN_1024_ to hPDLSCs cells via crystal violet staining and cytoskeleton staining (scale bar = 50 μm). (**E**) Migration of *sh-ITGB1*-hPDLSCs cells cultured on rhFN_1024_ at 0, 24 and 48 h (scale bar = 200 μm). (**F**) Quantitative analysis of the gap area of *sh-ITGB1*-hPDLSCs cells cultured on rhFN_1024_. (**G**) The CCK-8 method was used to detect the effects of different concentrations of rhFN_1024_ on the proliferation of *sh-ITGB1*-hPDLSCs after 24, 48 and 72 h. (**H**) Determination of the adhesion of rhFN_1024_ to *sh-ITGB1*-hPDLSCs cells via crystal violet staining and cytoskeleton staining (scale bar = 50 μm); *n* = 3, means ± SD. *P < 0.05, **P < 0.01, ***P < 0.01 vs. the control.

To investigate the effect of rhFN_1024_ on hPDLSCs by interacting with integrin β1, β1 was knocked down in hPDLSCs (Supplementary Figure S4). The CCK-8 experiments showed that rhFN_1024_, at concentrations ranging from 3.75 to 30 μg/ml, did not influence the proliferation of either hPDLSCs or *sh-ITGB1*-hPDLSCs (Figure 3C and G). Secondly, in the wound healing assay, for hPDLSCs, rhFN_1024_ at 12 μg/ml was a significant promoter of wound healing at 24 and 48 h, with a wound healing rate of 40% and 85%, respectively (Figure 3A and B, **P < 0.01, ***P < 0.001). However, for *sh-ITGB1*-hPDLSCs, low and high concentrations of rhFN_1024_ did not significantly promote wound healing at 24 h. After 48 h, the cell migration capability at concentrations of 6 and 12 μg/ml significantly increased, reaching 68% and 72%, respectively, and exhibiting a dose-dependent response (Figure 3E and F). To further analyse adhesion activity, we assessed hPDLSCs and *sh-ITGB1*-hPDLSCs cell adhesion to rhFN_1024_. Cells were incubated on each layer of the substrate for 4 h to facilitate adhesion, after which samples were fixed and stained to enable a comparative analysis of cytoskeletal development. hPDLSCs display a well-structured actin cytoskeleton. In contrast, cells subjected to integrin β1 knockdown exhibit a notable absence of organized actin structures (Figure 3D and H).

### rhFN_1024_ promoted the myofibroblast differentiation of hPDLSCs independent AGE injury

**Figure 4.**
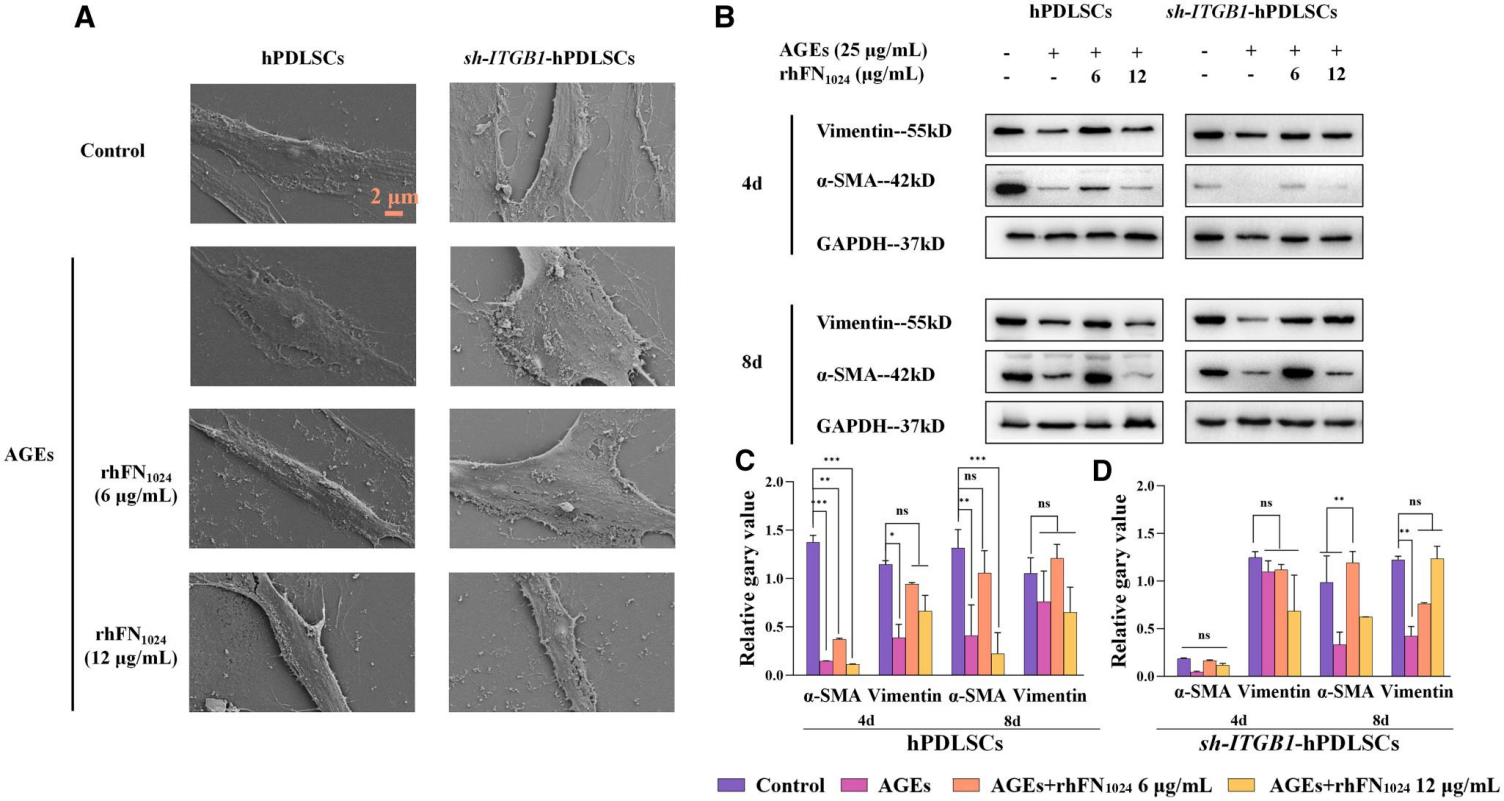
rhFN_1024_ promoted the myofibroblast differentiation of hPDLSCs with or without AGE induction. (**A**) The morphology of hPDLSCs and *sh-ITGB1*-hPDLSCs was observed with a scanning electron microscope (scale bar = 2 μm). (**B**) The expression of the fibrosis-related protein α-SMA and vimentin was analysed via Western blotting analysis. (**C**) Semi-quantitative analysis of α-SMA and vimentin expression on hPDLSCs. (**D**) Semi-quantitative analysis of α-SMA and vimentin expression on *sh-ITGB1*-hPDLSCs. *n* = 3, means ± SD. **P < 0.01, ***P < 0.001 vs. the control.

SEM showed that, among hPDLSCs, the cytosol experienced hypertrophy, and the cilia increased after 8 days of AGE induction. Moreover, the cells were long and flat with well-defined contours after the administration of 6 and 12 μg/ml of rhFN_1024_. The *sh-ITGB1*-hPDLSCs also showed hypertrophy after 8 days of AGE induction. However, the cells did not change significantly after the administration of 6 μg/ml of rhFN_1024_. But the cells changed to a long, flattened shape when the rhFN_1024_ concentration was increased to 12 μg/ml. The overall cell outline was not as well-defined as that of the normal hPDLSCs (Figure 4A). Based on the overall cell morphology, we speculate that rhFN_1024_ promotes the fibroblast differentiation of hPDLSCs, and ITGB1 participates in the promotion of fibroblast differentiation by rhFN_1024_.

From Figure 4B and C, in the hPDLSCs groups, on the fourth day, we observed a significant decrease in α-SMA after AGE injury and a slight upregulation of α-SMA when treated with 6 μg/ml rhFN_1024_. With continuous administration of rhFN_1024_ for 8 days, the expression of α-SMA was restored to a normal level.

On the fourth day post-injury by AGEs, *sh-ITGB1*-hPDLSCs exhibited a slight decrease in α-SMA and vimentin expression. Treatment with rhFN_1024_ led to increased expression of α-SMA. On the eighth day, α-SMA expression was significantly upregulated in the rhFN_1024_-treated groups (*P* < 0.01). The expression of vimentin did not show significant changes at day 8, regardless of whether in the hPDLSCs or *sh-ITGB1*-hPDLSCs groups (Figure 4B and D). Based on the protein expression levels, we inferred that rhFN_1024_ was able to promote the differentiation of hPDLSCs into myofibroblasts, thereby facilitating wound healing.

### hPDLSCs@rhFN_1024_ promoted wound healing in diabetic mice with full-thickness skin defects

**Figure 5.**
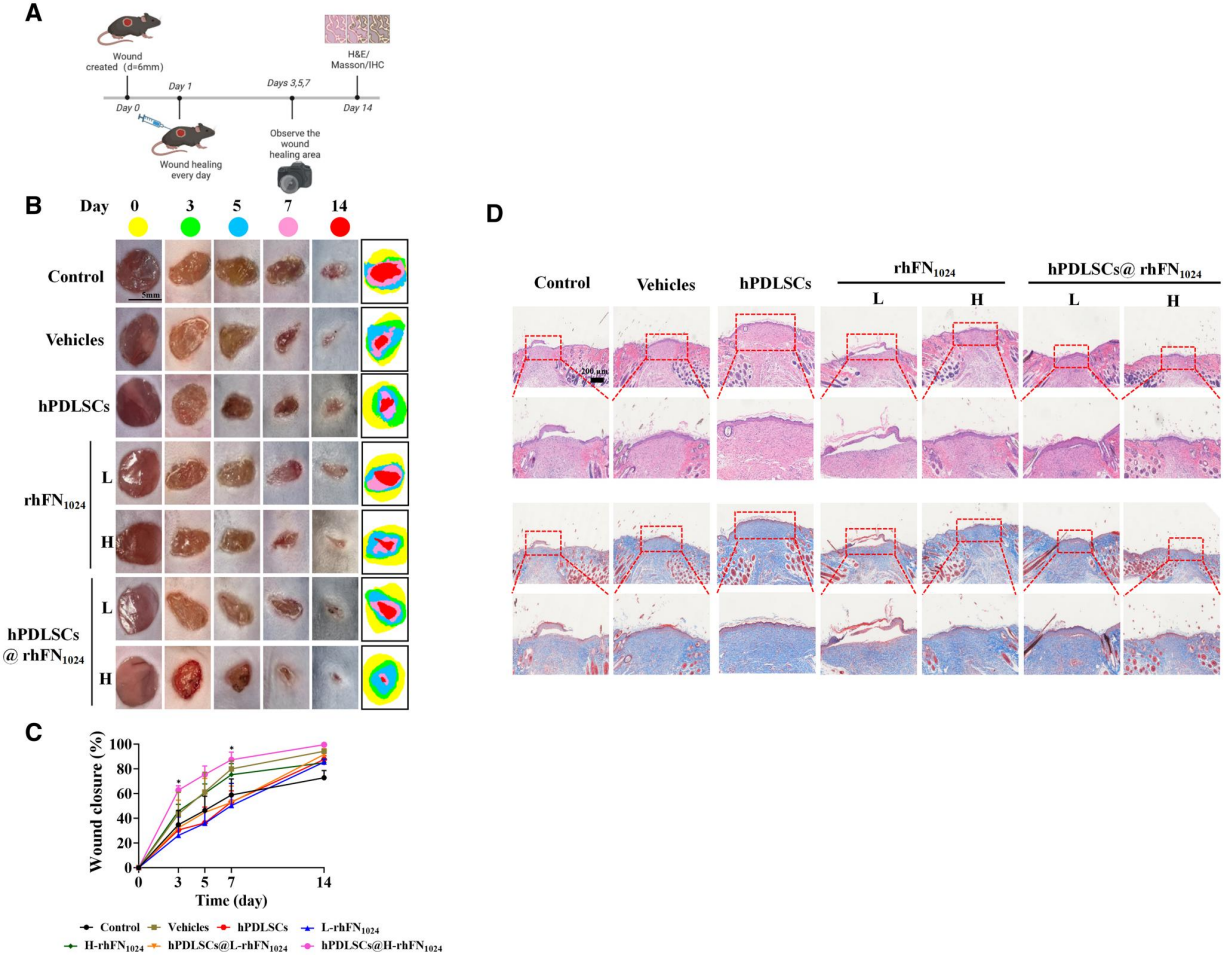
Evaluation of wound healing in a diabetic model *in vivo*. (**A**) The experimental process of modeling and treatments. (**B**) Wounds were photographed on days 0, 3, 5, 7 and 14. Traces of wound closure were found at 14 days (scale bar = 5 mm). (**C**) Wound closure rates of the seven groups. (**D**) H&E and Masson’s trichrome (MT) staining of wound tissues on day 14 of surgery (scale bar = 200 μm). *n* = 5, means ± SD. *P < 0.05 vs. the control.

Following the establishment of the full-thickness skin defect model, treatments were administered using hPDLSCs, rhFN_1024_ and hPDLSCs@rhFN_1024_ (Figure 5A). Three days after wounding, the hPDLSCs@H-rhFN_1024_ group exhibited a significantly higher wound healing rate (62.7% ± 3.63%) compared to other groups (*P* < 0.05), achieving 1.8 times the rate of the control group. On day 7, the hPDLSCs@H-rhFN_1024_ group achieved a wound healing rate of 87.39% ± 6.16% compared to the other groups (Figure 5B and C). The combination of hPDLSCs and rhFN_1024_ improved diabetic wound healing. Histological analysis was then performed to assess skin formation. Hematoxylin and eosin (H&E) staining showed that the wounds in the hPDLSCs@H-rhFN_1024_ group featured the smallest wound cross-sectional areas at 14 days and presented intact re-epithelialization and reduced traumatic inflammatory factors. The mice in the control group did not have intact re-epithelialization and contained large numbers of inflammatory cells (Figure 5D). The H&E staining results were consistent with the observed wound healing images, and the results confirmed that the combination of hPDLSCs and rhFN_1024_ significantly promoted wound healing. In the final stages of wound healing, collagen deposition is necessary to reconstruct the wound. Collagen deposition in the tissue was measured using Masson’s trichrome staining. As shown in Figure 5D, on day 14, the hPDLSCs@H-rhFN_1024_ group displayed stronger intensity near the wound compared to the control group, suggesting that collagen deposition was strongest in the wounds treated with H-rhFN_1024_ in combination with hPDLSCs and that the collagen arrangement was more tightly ordered compared to that in the rest of the groups.

### hPDLSCs@rhFN_1024_ reduced NF-κB/TNFα induced inflammation to promote wound healing in diabetic mice

**Figure 6.**
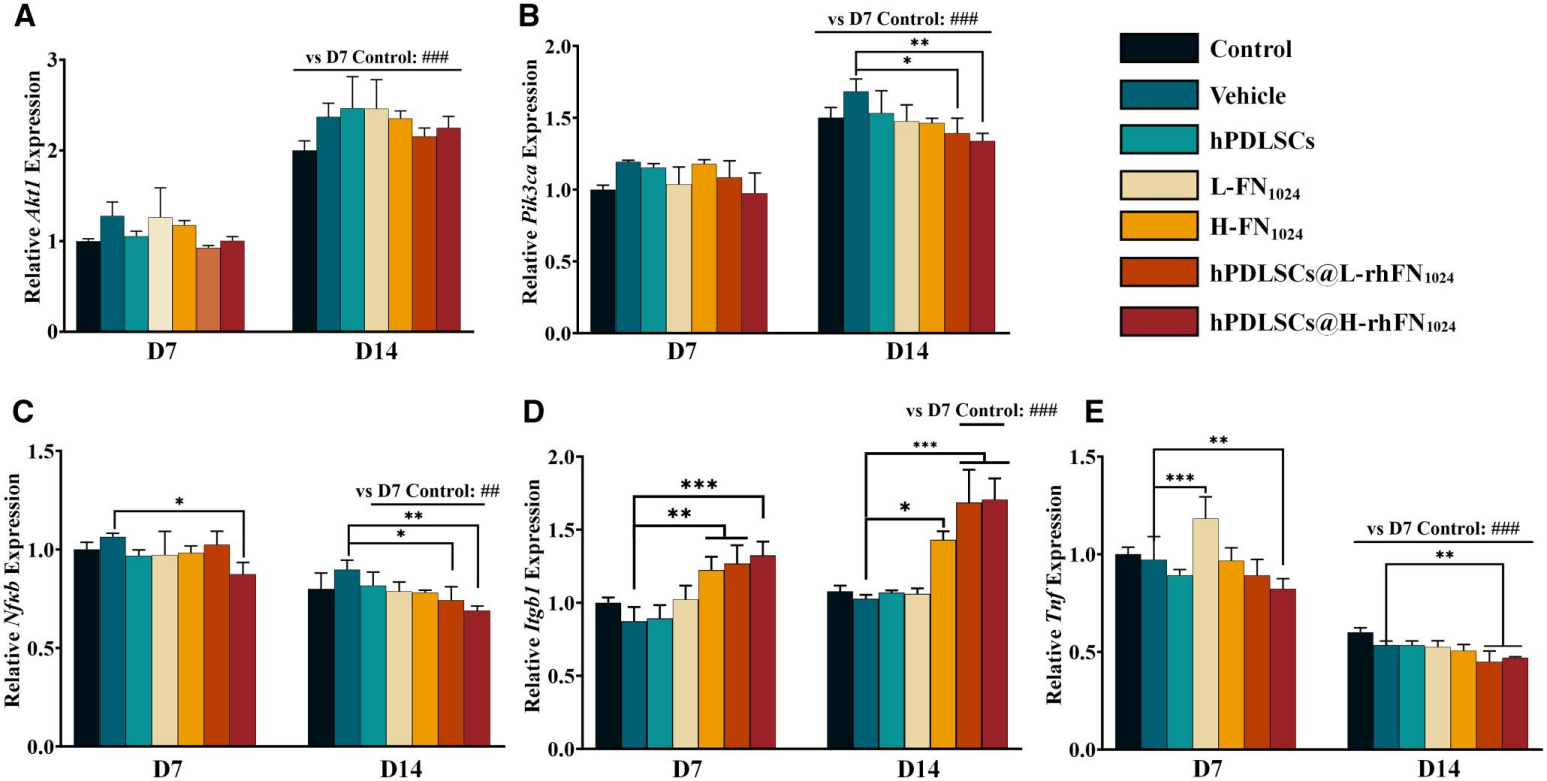
qRT-PCR was employed to quantitate the mRNA levels of (**A**) *Akt1*, (**B**) *Pik3ca*, (**C**) *Nfkb*, (**D**) *Itgb1* and (**E**) *Tnf* in animal tissues on days 7 and 14. *P < 0.05, **P < 0.01, ***P < 0.001 vs. the vehicle. ##*P* < 0.01, ###*P* < 0.001 vs. the day 7 control.

RT-qPCR was used to detect the mRNA expression levels of *Tnf*, *Itgb1*, *Pik3ca*, *Akt1* and *Nfkb* in the damaged skin collected on the seventh and 14th day. RT-qPCR analysis confirmed that on day 14, the expression levels of *Akt1* and *Pik3ca* genes were elevated, while those of *Nfkb* and *Tnf* genes were reduced relative to day 7. However, the mRNA expression of *Itgb1* was upregulated only in the H-rhFN_1024_ and hPDLSCs@rhFN_1024_ groups. In addition, the hPDLSCs@rhFN_1024_ group demonstrated a significant upregulation in *Itgb1* mRNA expression, along with a marked downregulation of *Pik3ca*, *Akt1*, *Nfkb* and *Tnf* expression levels when compared to the vehicle-treated group (Figure 6).

**Figure 7.**
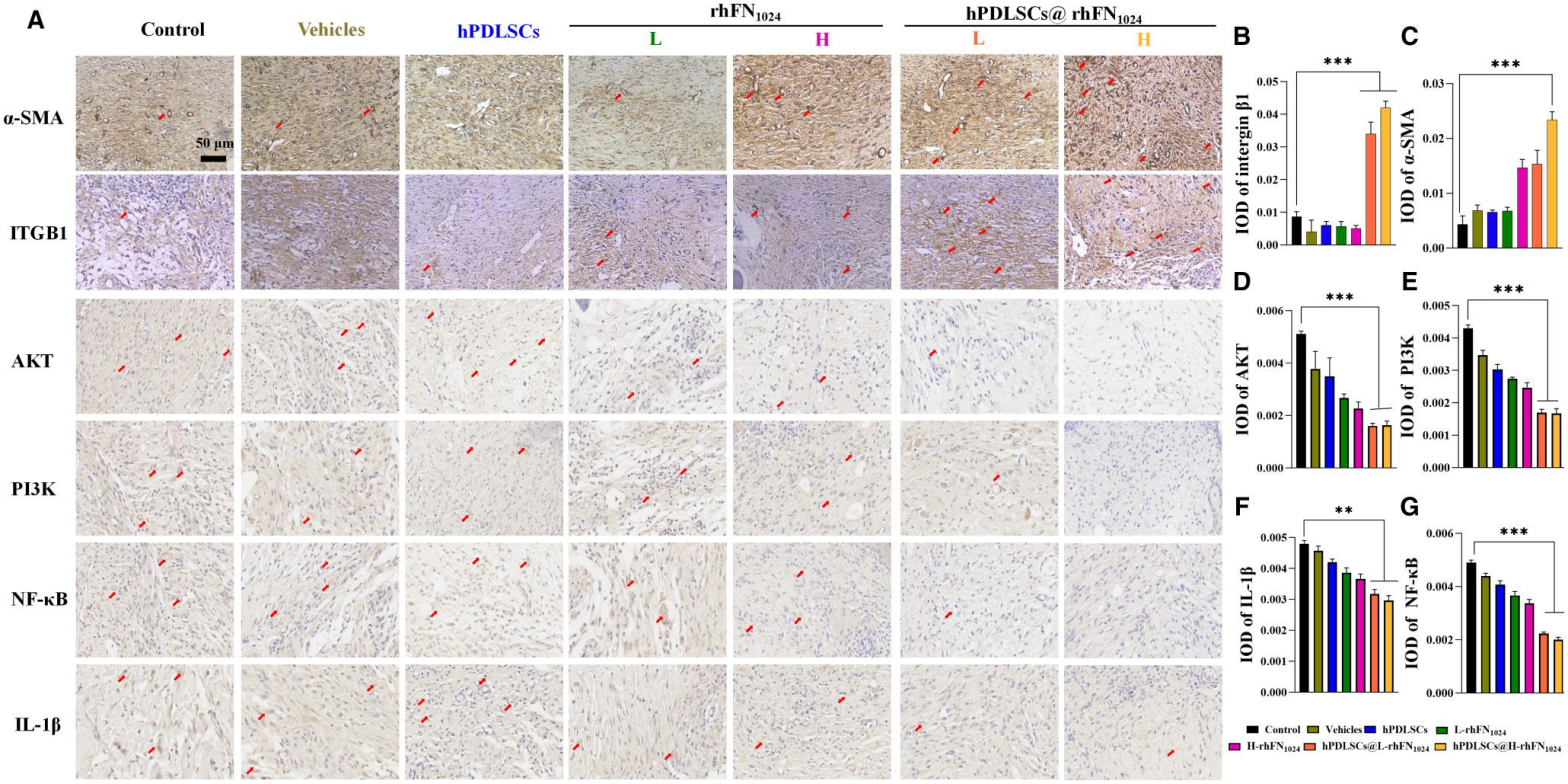
Immunohistochemical examination of α-SMA, integrin β1, AKT, PI3K, NF-κB and IL-1β expression. (**A**) Immunohistochemical labeling of α-SMA, integrin β1, AKT, PI3K, NF-κB and IL-1β-positive cells on day 14. The arrows indicate representative positive results of AKT, PI3K, NF-κB and IL-1β (scale bar = 50 µm). Semi-quantitative analysis of (**B**) α-SMA, (**C**) integrin β1, (**D**) AKT, (**E**) PI3K, (**F**) NF-κB and (**G**) IL-1β expressing cells at 14 days, measured using the Image J software. *n* = 3, mean ± SD, **P < 0.01, ***P < 0.001 vs. the control.

The expression levels of α-SMA, integrin β1, PI3K, AKT, NF-κB and IL-1β in the damaged skin samples collected on day 14 were quantitatively assessed using immunohistochemical analysis (Figure 7). The hPDLSCs@rhFN_1024_ group demonstrated a significant upregulation in the expression of integrin β1 (*P* < 0.001). The hPDLSCs@L-rhFN_1024_ and hPDLSCs@H-rhFN_1024_ groups exhibited a significant reduction in the expression of PI3K, AKT, NF-κB and IL-1β (*P* < 0.01). As shown in Figure 7A, the key factors PI3K, AKT and NF-κB were significantly reduced in the hPDLSCs@rhFN_1024_ group compared to those in the vehicle-treated group. In the hPDLSCs@L-rhFN_1024_ group, the expression of PI3K, AKT and NF-κB was significantly lower than in the vehicle-treated group, with no significant difference from the control group. There was no significant difference in the expression of PI3K, AKT and NF-κB between the L-rhFN_1024_ and H-rhFN_1024_ groups. The inflammatory factor IL-1β showed the same trend as above.

## Discussion

ECM structural proteins are ideal functional protein matrix materials and have great application prospects in the medical, military and green manufacturing fields as functional protein matrix materials [23, 24]. During tissue repair, the reconstruction of the ECM depends on the correct assembly of ECM structural proteins such as FN and collagen. The perfect ECM is achieved through a strict hierarchical assembly pattern, which begins with the deposition of FN filaments on the cell surface. Thus, FN plays an important role in the communication between cells and the ECM [25]. Integrin–FN interactions determine cell adhesion and migration and are involved in cellular differentiation [24]. The rhFN_1024_ constructed in this study did not contain glycosylation sites or use *E. coli* as the expression host. The rhFN_1024_ in this study also did not involve post-translational modification of the protein. Previous studies suggest that glycosylation modification of FN is more helpful for its physiological functions [26, 27]. A recent study has demonstrated that the glycosylation of fibronectin (FN) inhibits the recruitment of c-Src to vascular endothelial growth factor receptor 2 (VEGFR-2) via the receptor for RAGE. Notably, treatment with an anti-RAGE antibody restored VEGF-induced phosphorylation of VEGFR-2, Akt and extracellular signal-regulated kinase 1/2 (ERK1/2), as well as endothelial cell migration, proliferation and tube formation. In other words, the glycosylation of FN markedly suppresses VEGF-induced neovascularization and further exacerbates the impairment of angiogenesis in diabetic ischemic disease [28]. In this article, a non-glycosylated rhFN_1024_ was constructed to reconstruct the ECM structure and displayed relatively satisfactory results.

Integrin β1 is present on the surfaces of major cells in the temporary ECM in damaged skin. In the uninjured epidermis, integrin β1 is expressed at low levels but is upregulated during wound healing [29]. The role of FN in wound healing is inextricably linked to that of integrin β1 due to the integrin-binding domain in FN [30]. Wang *et al.* reported that during keratinocyte migration and cutaneous wound repair, integrin β1 plays a key role [29]. Once FN binds to integrins, it transmits various signals to regulate key cellular processes.

In hPDLSCs, the expression and activity of integrin β1 are crucial for cell adhesion, migration, proliferation and differentiation. It transmits signals into hPDLSCs through interactions with the ECM, regulating the organization of the cytoskeleton, gene expression and cell fate determination. hPDLSCs have powerful myofibroblast differentiation properties during *in vitro* expansion [15, 31]. A recent report showed that dermal α-SMA myofibroblasts facilitate skin wound healing through integrin β1, without relying on type I collagen synthesis [32]. These myofibroblasts could migrate to the wound surface in addition to producing various growth factors and ECM components [33], which was beneficial for the healing of diabetic foot ulcers to reduce the amputation rate. Moreover, integrin β1 plays a crucial role in modulating the immune response and inflammation in hPDLSCs, essential for periodontal tissue repair and regeneration. Therefore, studying the function and regulatory mechanisms of rhFN_1024_ on integrin β1 in hPDLSCs can help to deepen the understanding of the biological characteristics of rhFN_1024_ and hPDLSCs. Our lab concentrates on the application of hPDLSCs in tissue reconstruction and has reported a series of papers to reveal their potential [22, 34, 35].

To determine the reliability of rhFN_1024_ binding with integrin β1 on hPDLSCs, we predicted the fibronectin structure and the position of FN_1024_ in fibronectin. As the crystal structure of fibronectin was not available in the PDB, we had to predict its structure. The molecular weight of the fibronectin subunits ranges from 220 to 250 kDa, and it comprises over 2000 amino acids [36]. Consequently, the predicted structural configuration of fibronectin is inadequate to facilitate further molecular docking with integrin β1, hampering the undesired ‘spatial site blocking’ effect. Fortunately, high-resolution crystal structures of FN_12-14_ and ITGB1 are recorded in the PDB, enabling us to simulate the interactions between rhFN_1024_ and ITGB1 utilizing information sourced from https://www.ebi.ac.uk/pdbe/pisa/. Our findings revealed an interaction interface of 892.5 Å^2^ and a free energy of −1.6 Δ*G*, suggesting a stable complex formation between rhFN_1024_ and ITGB1. This observation underscores the potential significance of integrin β1 as a crucial functional anchor for rhFN_1024_. The results were further confirmed by CETSA and shRNA technology.

In the cytological experiments, the expression of ITGB1 in hPDLSCs was downregulated in the AGEs environment. Meanwhile, when we silenced integrin β1 using shRNA, the proliferation and differentiation of hPDLSCs were inhibited. However, rhFN_1024_ still promoted hPDLSC myofibroblast differentiation, especially after injury with AGEs. In the cell migration assays, we observed that rhFN_1024_ (12 μg/ml) had a significant wound healing promotion effect on hPDLSCs at 48 h (the wound healing rate of rhFN_1024_ was about 85% vs. 64% for the blank). After *sh-ITGB1*-hPDLSCs were treated with rhFN_1024_ for 48 h, there was no significant increase in the wound healing rate (blank was 58%, 6 μg/ml of rhFN_1024_ was 68% and 12 μg/ml of rhFN_1024_ was 72%). The results indicated that integrin β1 significantly influences the efficacy of rhFN_1024_. After being damaged by AGEs, the morphology of hPDLSCs and *sh-ITGB1*-hPDLSCs became hypertrophic, as observed via SEM, and *sh-ITGB1*-hPDLSCs were more deformed. When hPDLSCs were treated with rhFN_1024_, the morphology recovered. However, rhFN_1024_ had little effect on *sh-ITGB1*-hPDLSCs. Therefore, rhFN_1024_ protects hPDLSCs against AGE injury, a process closely associated with integrin β1.

The surface markers of myofibroblasts (α-SMA) and fibroblasts (vimentin) were tested using WB. The study found a significant reduction in α-SMA levels in hPDLSCs following AGE injury. When treated with rhFN_1024_ (6 μg/ml), the expression of α-SMA was upregulated after 8 days. In the *sh-ITGB1*-hPDLSCs, the expression of α-SMA was limited in all experimental groups after 4 days. However, on the eighth day, α-SMA expression showed partial recovery. Compared to the untreated *sh-ITGB1*-hPDLSC group, rhFN_1024_ (6 μg/ml) intervention resulted in a significantly higher expression of α-SMA (*n* = 3, *P* < 0.01). The restoration of α-SMA and vimentin expression in *sh-ITGB1*-hPDLSCs at day 8 was unexpected. We speculate that the differentiation of hPDLSCs may be much more complex than currently understood and may rely on other factors in addition to ITGB1. Initially, ITGB1 showed positive effects. However, as time progressed, the effects of ITGB1 were replaced with other processes that are not yet fully understood. Furthermore, the vimentin level did not change significantly throughout the entire experiment, which means that the dedifferentiated phenotype was not a fibroblast phenotype.

**Figure 8.**
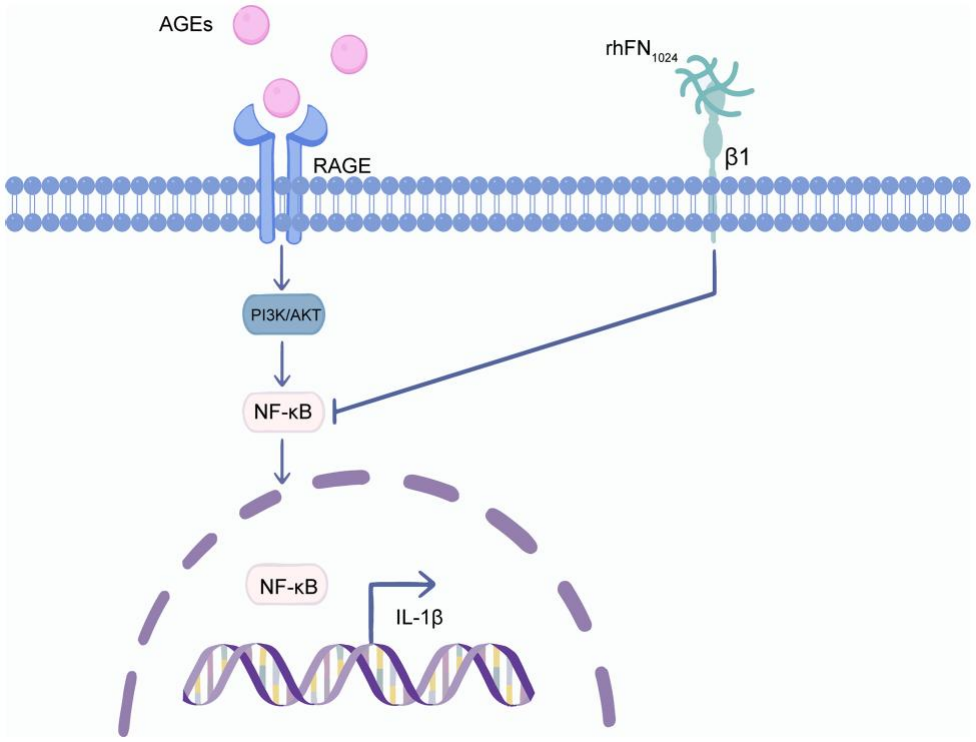
A schematic illustration of the potential mechanism based on the evidence presented in the present study.

The difficulty in healing diabetic wounds is primarily due to chronic hyperglycemia and neuropathy of the wound induced by AGEs [32–34]. In this study, type 1 diabetes was induced in mice via STZ. Using whole-skin injury in the diabetic mice, the hPDLSCs@H-rhFN_1024_ group had a significantly higher recovery rate (87.39 ± 6.16%) than the rest of the groups at day 7 (*P* < 0.05). On day 14 of the H&E staining analysis, the hPDLSCs@H-rhFN_1024_ group exhibited the smallest wound cross-sectional area, significantly reduced inflammatory factors and complete re-epithelialization compared to other groups. Masson’s staining showed greater collagen deposition and a denser and more ordered arrangement in the hPDLSCs@H-rhFN_1024_ group. The expression of α-SMA, a surface marker of myofibroblasts, was highest in the hPDLSCs@H-rhFN_1024_ group, as was the expression of integrin β1. hPDLSCs@L-rhFN_1024_ also showed a similar phenomenon. Thus, the combination of hPDLSCs with rhFN_1024_ accelerates diabetic wound healing by differentiating hPDLSCs into myofibroblasts via integrin β1. Diabetic patients exhibit a heightened risk of wound infections due to impaired healing mechanisms. However, the antibacterial efficacy of the rhFN_1024_ hydrogel has yet to be comprehensively validated, necessitating further investigation into its therapeutic potential for infected wound healing. Additionally, the clinical application of hPDLSCs therapy faces significant challenges, including limited accessibility to stable cell sources, low *in vitro* expansion capacity, pronounced cellular heterogeneity and poor post-transplantation survival rates. Immune rejection further complicates its translational viability. To further clarify the roles of rhFN_1024_ and ITGB1 in the healing of diabetic wounds, we delved into the mechanisms of AGE-induced injury. It has been documented that AGEs interact with RAGE via the PI3K/AKT/NF-κB signaling pathway, thereby exacerbating inflammatory responses. The PI3K/AKT pathway is a crucial signaling cascade that significantly influences cell proliferation, differentiation and migration. We utilized qPCR and immunohistochemical analyses and discovered that integrin β1, PI3K and AKT were significantly upregulated, while NF-κB and TNFα were notably downregulated, and IL-1β was remarkably downregulated. This indicates that although AGEs increased the expression of PI3K/AKT, the rhFN_1024_ hydrogel carrying hPDLSCs synergistically reduced the levels of inflammatory factors by suppressing the expressions of NF-κB, TNFα and IL-1β (Figure 8). Whether the activation of ITGB1 also affects the expression of cellular inflammatory factors through other related pathways warrants further investigation.

## Conclusion

In conclusion, our rhFN_1024_ yield was above 2 g/l, and it did not influence hPDLSCs proliferation. The ability of rhFN_1024_ to bind with integrin β1 was demonstrated through computer simulations and CETSA. rhFN_1024_ promoted the migration and adhesion of hPDLSCs by binding to integrin β1 and assisted hPDLSC differentiation into myofibroblasts in an AGE-induced high-glucose environment. In the full-thickness skin defect diabetic mice model, the hPDLSCs@H-rhFN_1024_ group exhibited an enhanced wound healing rate compared to other groups. This result suggests that the combination of hPDLSCs and rhFN_1024_ facilitated wound healing in a high-glucose environment.

## Supplementary Material

rbaf027_Supplementary_Data
