# Supplementary material for: Recombinant human fibronectin segment (rhFN1024) hydrogel carried hPDLSCs to repair diabetic trauma by activated NF-κB signaling pathway
Source: Regen Biomater. 2025 May 15;12:rbaf027. doi: 10.1093/rb/rbaf027 (PMC12119132; doi:10.1093/rb/rbaf027)
Supplement: rbaf027_Supplementary_Data [file rbaf027_supplementary_data.zip › Proofreading reply.docx]

Dear Editorial Office,

Thank you for sending the proof of our manuscript entitled “Recombinant human fibronectin segment (rhFN1024) hydrogel carried hPDLSCs to repair diabetic trauma by activated NF-κB signaling pathway” (Manuscript ID: RB-2025-004.R1). We have carefully reviewed and made adjustments or corrections prior to the revised manuscript. Below is a summary of the changes made:

1. **Typographical Errors**: The typos such as "fugure" have been corrected. Except for the percent sign (%) and temperature symbols (℃ or ℉), a space have been added after all numbers before the unit.
2. **Figure/Table Revisions**: Except for the graphical abstract, all figures have been numbered.
3. **Affiliation Deletion:** Removed affiliation " The First Affiliated Hospital of Jinan University " from " Affiliation 4”.

We hope that the newer revised manuscript can meet the requirements of the journal publication. We appreciate the efficient handling of our manuscript by your team. Please contact us if further clarifications are needed.

Sincerely,

Qi Xiang, Ph.D.,

Institute of Biomedicine and Guangdong Provincial Key Laboratory of Bioengineering Medicine, Jinan University,

Guangzhou, GZ, China.

E-mail: txiangqi@jnu.edu.cn

Fax:020-85565109

Phone no:13928832573
