# Supplementary material for: Recombinant human fibronectin segment (rhFN1024) hydrogel carried hPDLSCs to repair diabetic trauma by activated NF-κB signaling pathway
Source: Regen Biomater. 2025 May 15;12:rbaf027. doi: 10.1093/rb/rbaf027 (PMC12119132; doi:10.1093/rb/rbaf027)
Supplement: rbaf027_Supplementary_Data [file rbaf027_supplementary_data.zip › Supplementary data-0410.docx]

**Supplementary Figures**


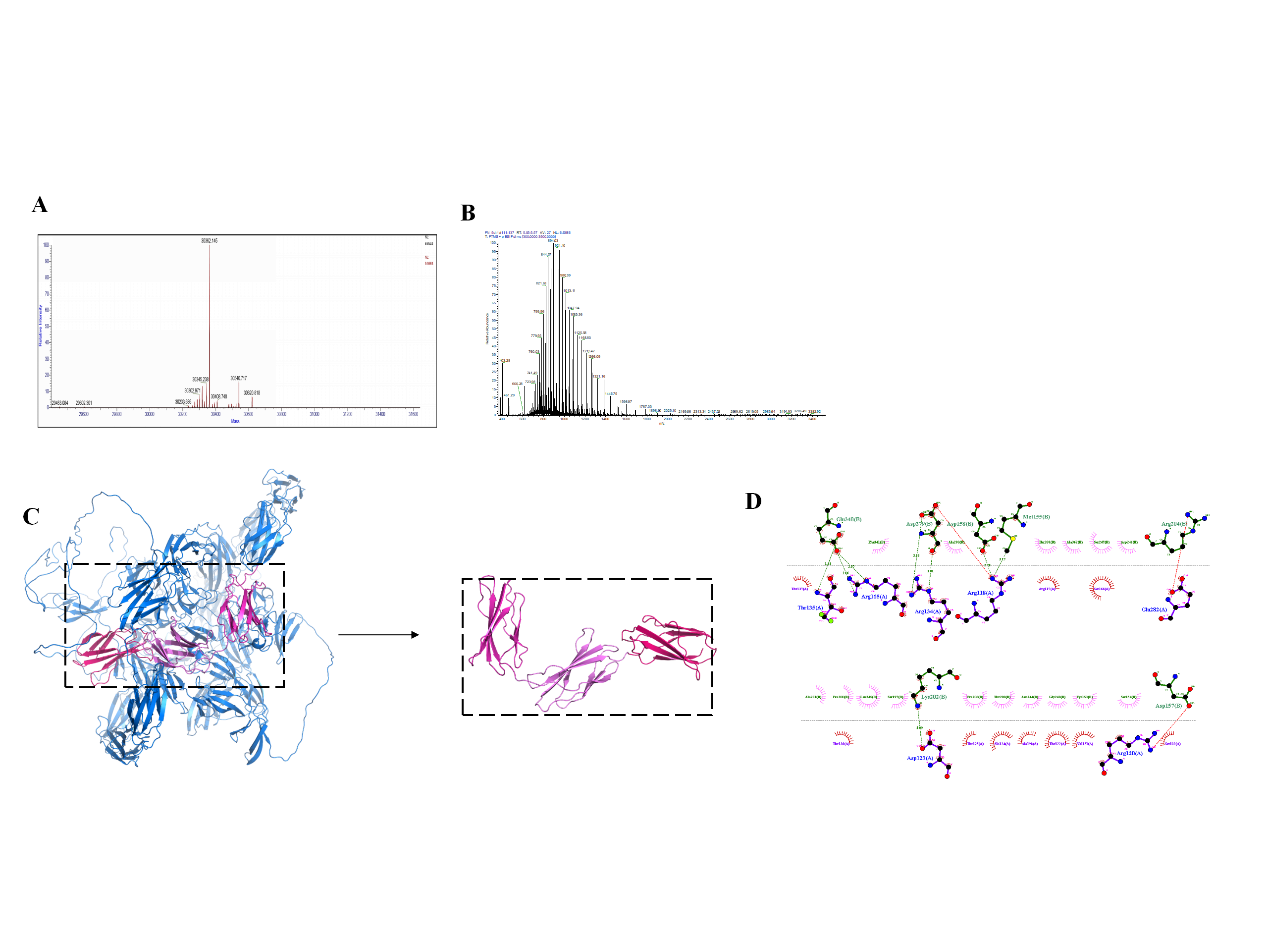


**Figure S1.** Precise molecular weight analysis and molecular docking. (**A**) rhFN_1024_ by LC-MS/MS (**B**) rhFN_1024_ peptide fingerprinting. (**C**) 3D structure of rhFN_12-14_ and its position in the 3D structure of fibronectin. (D) 2D plot of the molecular interaction of rhFN-Integrin β1


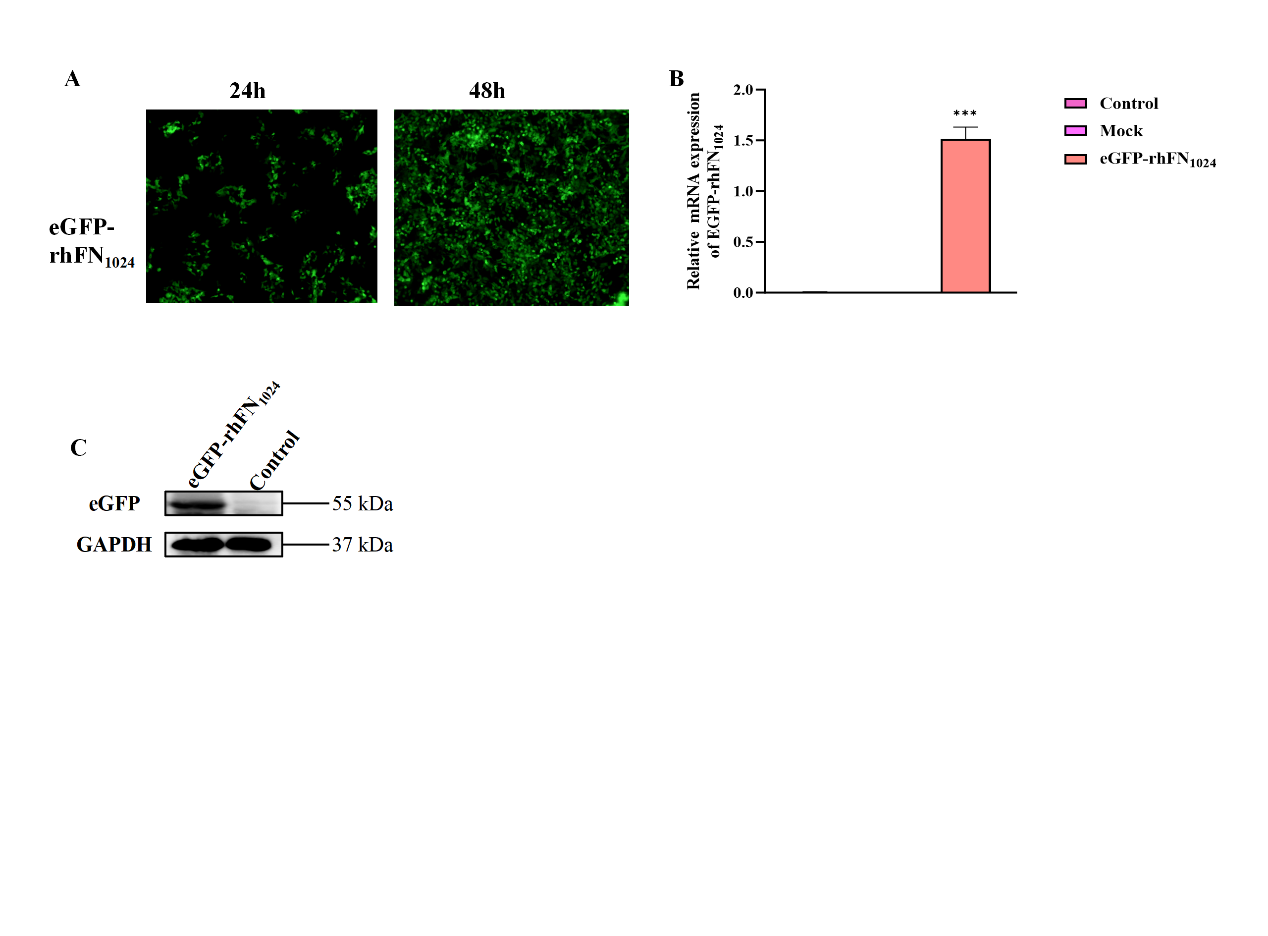


**Figure S2.** eGFP-rhFN_1024_ was overexpressed in 293T cells. (**A**) Representative images of eGFP-rhFN_1024_^+^ cells. (**B**) qRT-PCR was employed to quantitate the mRNA levels of eGFP-rhFN_1024_ in 293T cells. (**C**) The expression of the eGFP-rhFN_1024_ protein was analyzed via Western blotting analysis. n=3, mean ± SD, ***, *P<0.001* *vs*. the control


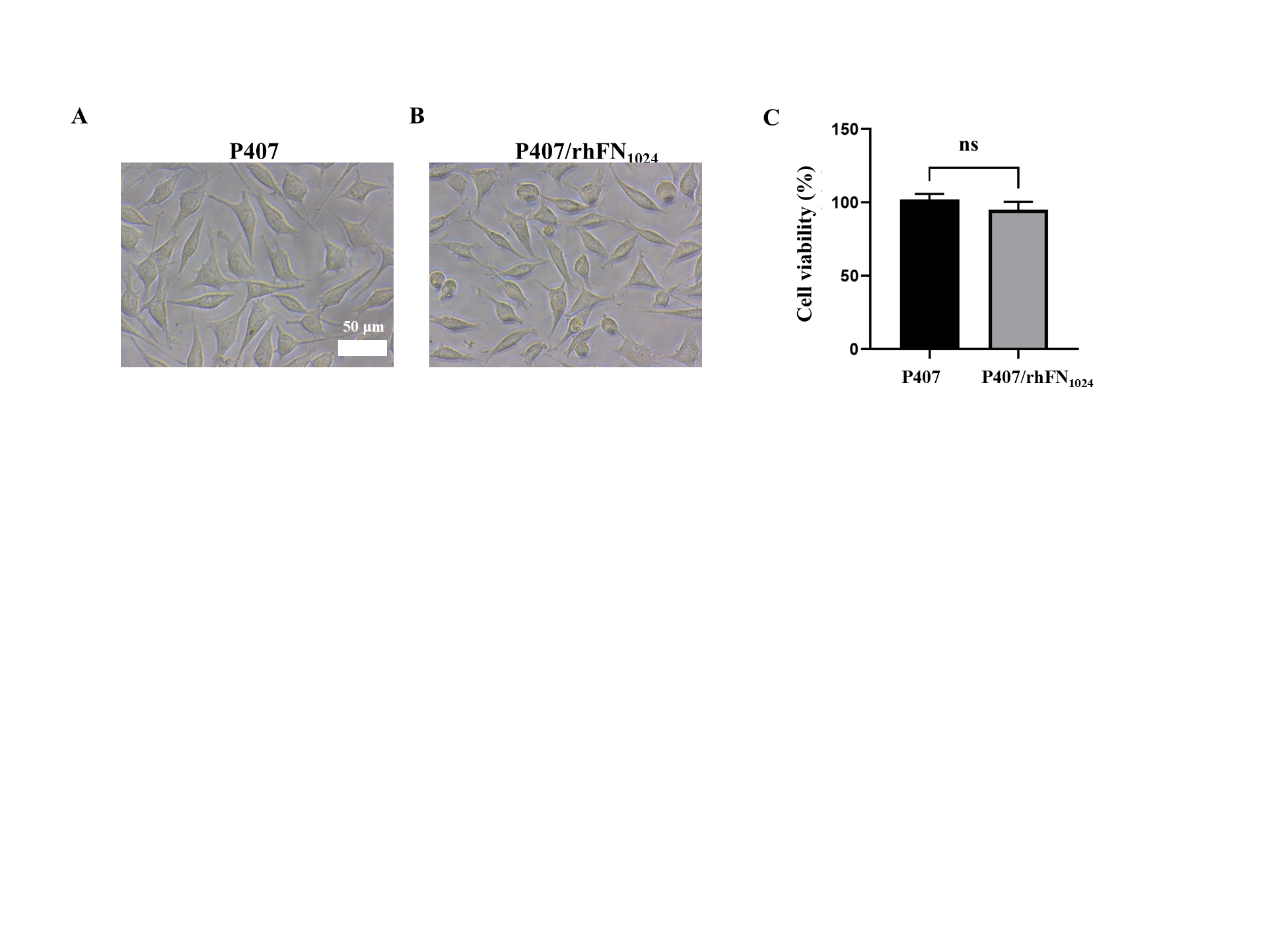


**Figure S3.** The biocompatibility of P407/rhFN_1024_. (**A**) Representative images of L929 cells treated with P407 hydrogel. (**B**) Representative images of L929 cells treated with P407/rhFN_1024_ hydrogel. (**C**) Cell viabilities of L929 cells treated with P407 hydrogel or P407/rhFN_1024_. n=3, mean ± SD, ns: no significance difference *vs* P407 hydrogel.


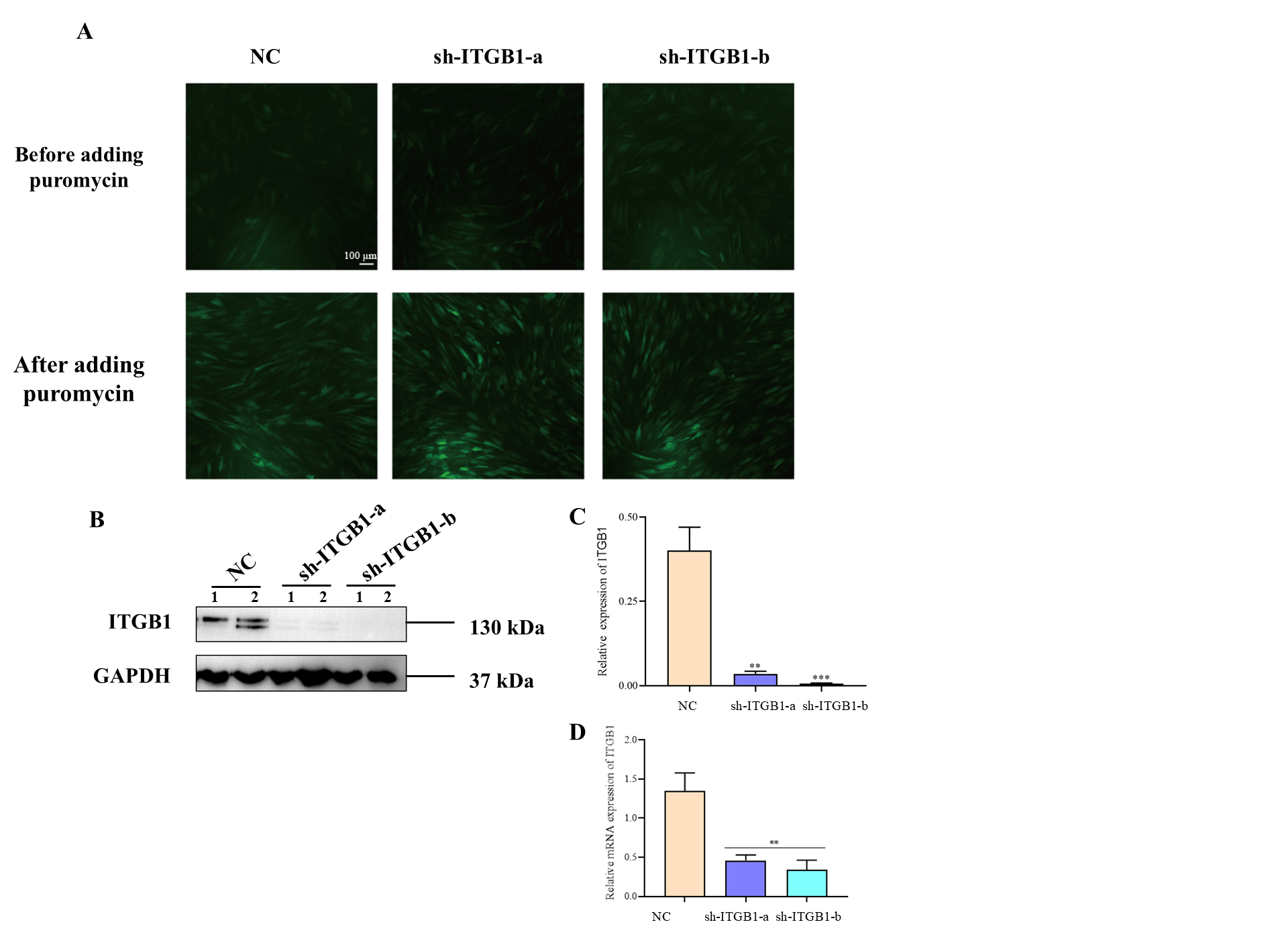


**Figure S4.** *shRNA-ITGB1* interfered with integrin β1 in hPDLSCs. (**A**) Representative images of sh-ITGB1^+^ cells. (**B**) The expression of the integrin β1 protein was analyzed via Western blotting analysis and (**C**) semi-quantitative analysis. (**D**) qRT-PCR was employed to quantitate the mRNA levels of integrin β1 both in hPDLSCs and sh-ITGB1-hPDLSCs. n=3, mean ± SD, **, *P<0.01*, ***, *P<0.001* *vs*. the NC group. NC, Normal Control.

**Supplementary table**

**Table S1**. shRNA Target Sequence

| shRNA | Target Sequence |
| --- | --- |
| ITGB1-a | 5ʹ-CCAAATCATGTGGAGAATGTA-3ʹ |
| ITGB1-b | 5ʹ-GCATATCTGGAAATTTGGATT-3ʹ |
